# Unique food-entrained circadian rhythm in cysteine414-alanine mutant mCRY1 transgenic mice

**DOI:** 10.1007/s41105-016-0050-1

**Published:** 2016-01-29

**Authors:** Satoshi Okano, Akira Yasui, Kiyoshi Hayasaka, Osamu Nakajima

**Affiliations:** Research Center for Molecular Genetics, Institute for Promotion of Medical Science Research, Faculty of Medicine, Yamagata University, Yamagata, 990-9585 Japan; Division of Dynamic Proteome in Cancer and Aging, Institute of Development, Aging and Cancer, Tohoku University, Sendai, 980-8575 Japan; Department of Pediatrics, Faculty of Medicine, Yamagata University, Yamagata, 990-9585 Japan; Miyuki-Kai Hospital, Kaminoyama, 999-3161 Japan

**Keywords:** Food-entrainable oscillator, Circadian clock, Nonphotic zeitgeber, Restricted feeding cycle, Food-anticipatory activity, Suprachiasmatic nucleus, Interneuronal coupling

## Abstract

Food availability is a potent environmental cue that directs circadian locomotor activity in rodents. Daily scheduled restricted feeding (RF), in which the food available time is restricted for several hours each day, elicits anticipatory activity. This food-anticipatory activity (FAA) is controlled by a food-entrainable oscillator (FEO) that is distinct from the suprachiasmatic nucleus (SCN), the master pacemaker in mammals. In an earlier report, we described generation of transgenic (Tg) mice ubiquitously overexpressing cysteine414-alanine mutant mCRY1. The Tg mice displayed long locomotor free-running periods (approximately 28 h) with rhythm splitting. Furthermore, their locomotor activity immediately re-adjusted to the advance of light–dark cycles (LD), suggesting some disorder in the coupling of SCN neurons. The present study examined the restricted feeding cycle (RF)-induced entrainment of locomotor activity in Tg mice in various light conditions. In LD, wild-type controls showed both FAA and LD-entrained activities. In Tg mice, almost all activity was eventually consolidated to a single bout before the feeding time. The result suggests a possibility that in Tg mice the feeding cycle dominates the LD cycle as an entrainment agent. In constant darkness (DD), wild-type mice exhibited robust free-run activity and FAA during RF. For Tg mice, only the rhythm entrained to RF was observed in DD. Furthermore, after returning to free feeding, the free-run started from the RF-entrained phase. These results suggest that the SCN of Tg mice is entrainable to RF and that the mCRY1 mutation alters the sensitivity of SCN to the cycle of nonphotic zeitgebers.

**Electronic supplementary material:**

The online version of this article (doi:10.1007/s41105-016-0050-1) contains supplementary material, which is available to authorized users.

## Introduction

In mammals, the suprachiasmatic nucleus (SCN) is the master pacemaker that generates circadian locomotor behavior. Entrainment of the body clock to the light–dark cycle (LD) is a prime function of the SCN. Entrainment of circadian rhythms to LD is designated as photic entrainment. External photic time cues are perceived in the retina, and are transferred to the SCN; thereby the SCN clock can be adjusted to the environmental light conditions [1]. In each SCN neuron, the molecular clock mechanisms are operating. The mammalian molecular clockwork consists of a transcriptional–translational negative feedback loop (TTFL). Products of four clock gene families are involved in the main loop of TTFL: *Period* (*Per*), *Cryptochrome* (*Cry*), *Clock*, and *Bmal* [1]. Cryptochrome proteins (CRYs) play indispensable roles as inhibitive components in the TTFL [1, 2].

Interneuronal communication among SCN neurons, which is also designated as coupling, plays important roles in generating robust circadian rhythm of SCN as a whole, and confers stability against perturbation factors such as nonphotic cues [3, 4]. For instance in the case in which interneuronal communication among SCN neurons is inhibited pharmacologically, SCN tissues become more sensitive to temperature cues than the intact SCN [5]. In harmony with these findings, results of a mathematical modeling study demonstrated that coupling-induced rigidity in the SCN filters environmental noise to create a robust circadian system [6]. That coupling is mediated by various means such as neuropeptide-mediated signaling pathway and gap-junction [4]. Regarding neuropeptides, arginine vasopressin (AVP) and vasoactive intestinal peptide (VIP) are known to play key roles in the coupling of SCN neurons [3, 4]. Various mice having deficient coupling mechanisms have been reported. For instance, mice which lack vasopressin receptors V1a and V1b (*V1a*^−*/*−^*V1b*^−*/*−^ mice) exhibit abnormal entrainment behavior: their bout of locomotor activity shifts immediately in response to a shift of LD [7]. AVP neuron specific *Bmal1* deficient (*Avp*-*Bmal1*^−*/*−^) mice also re-entrain faster than wild-type controls after a phase advance in LD [8]. Furthermore, *Avp*-*Bmal1*^−*/*−^ mice and Ca^2+^/calmodulin-dependent protein kinase II alpha knock-in mice show rhythm splitting-like behaviors in constant darkness (DD) [8, 9].

In an earlier study, we created the mice overexpressing the mutant mCRY1 in which cysteine414 was replaced with alanine (previously we labeled the mutant protein CRY1-AP, but hereinafter we designate it as C414A mCRY1 and we designate the transgenic mice Tg mice). A recent structural–biological study uncovered the molecular implications of the role of cysteine414: cysteine414 of mCRY1 located on the surface of interaction with mPER2 forms an intermolecular zinc-binding site [10]. Actually, Tg mice display unusual circadian rhythms by which their locomotor free-running periods were long (about 28 h) with rhythm splitting [11]. Additionally, they readjust immediately to the 6-h phase advance of LD [11]. These phenotypic features common to the mice harboring SCN-neuronal coupling defect described above and to our Tg mice demonstrate the possibility that inter-neuronal communications in the SCN might be disturbed in Tg mice to some degree.

The daily scheduled restricted feeding cycle (RF), by which the food availability time is restricted for several hours per day, elicits anticipatory activity in rodents [12]. This food-anticipatory activity (FAA) is known to be controlled by a food-entrainable oscillator (FEO) that is regarded as distinct from the light-entrainable oscillator of SCN. The generation of FAA is independent of the circadian clock of SCN because it appears in animals whose SCN was destroyed [12]. The anatomical location and the molecular timekeeping mechanism of the FEO remain elusive [12]. Although the molecular clock in several peripheral tissues outside the SCN is entrained to RF, the SCN clock is fundamentally not entrained to RF [12] except in some particular cases. The most evident case is CS mice, in which SCN clock and locomotor rhythm entrain to RF under DD [13]. The mechanism by which the SCN entrains in the mice remains unknown.

Judging from the findings explained above, we inferred that the SCN of Tg mice might display anomalous entrainment behaviors under nonphotic zeitgeber cycles and that they might be an attractive model for studying unknown characteristics of the SCN. Therefore, in this study we examined RF-entrained circadian rhythm in Tg mice to elucidate the unique aspects of timekeeping and entrainment mechanism of oscillators of the mice. We observed locomotor activities of Tg mice under RF and various light conditions.

## Materials and methods

### Animals

We used the high expression line (H line) of Tg mice [11] and their littermates of wild-type mice having no transgene (WTs) for experiments. To maintain the Tg line, mice of BDF1 strain were used for the crossing. Because male Tg mice exhibit diabetes symptoms [14, 15], experimental analysis and interpretation are somewhat difficult. Therefore, female mice were used (6–15 weeks of age at the beginning of the experiment) for analyses of locomotor activities. In all the experiments, animals were treated in accordance with the Yamagata University guidelines.

### Measurement of wheel-running rhythms

Measurement of wheel-running activity and analysis of data are described in our previous report [11]. The mice were maintained on a 12-h light (approximately 150 lux, white light): 12-h dark (LD 12:12) cycle or a LD 14:10 cycle under ad libitum feeding for basically at least 3 weeks and were then transferred to the RF regime in designated LD conditions. For the LD 14:10 condition, lights were on during 07:00–21:00 of laboratory time. Before starting RF schedules, approximately 1 day of fasting (the exact time is described in the Results section) was done. For RF regimes under LD cycles, after the termination of RF schedules, fasting was also done for a period of time (see “Results”). During the RF regimes, food was available only 4 h per day with fixed onset and offset of meal times. Food pellets were given and taken away from the cage by hand. For phase-shifting experiments, the mice entrained to the first RF cycle for 8 days were subjected to a 4.5-h advance of the RF cycles. For RF experiments under constant darkness (DD) the exchange of food pellets was performed using an infrared viewer to avoid light exposure to mice.

### Quantification of mRNA in the liver using Real-Time PCR

Male mice (7–10 weeks of age) were synchronized to a LD12:12 cycle for at least 2 weeks under ad libitum feeding before tissue collection. Liver samples were collected at Zeitgeber time (ZT) 8 and 20. Each sample was immediately frozen in liquid nitrogen and stored at –80 °C until RNA extraction. The cDNA of the liver of mice used in the experiments is obtained as described in our previous report [11]. Real-time PCR analysis was conducted as described in an earlier report [15]. Results are expressed as relative values with respect to mHprt levels, as described earlier. Relevant primer sets are presented in Supporting Information.

### Statistical analysis

For each experiment, a *t* test was used to compare the mean values. Differences between means were inferred as significant for *P* < 0.05. Data are presented as mean ± SE.

## Results

### Food-entrained circadian rhythm in LD 12:12

**Fig. 1:**
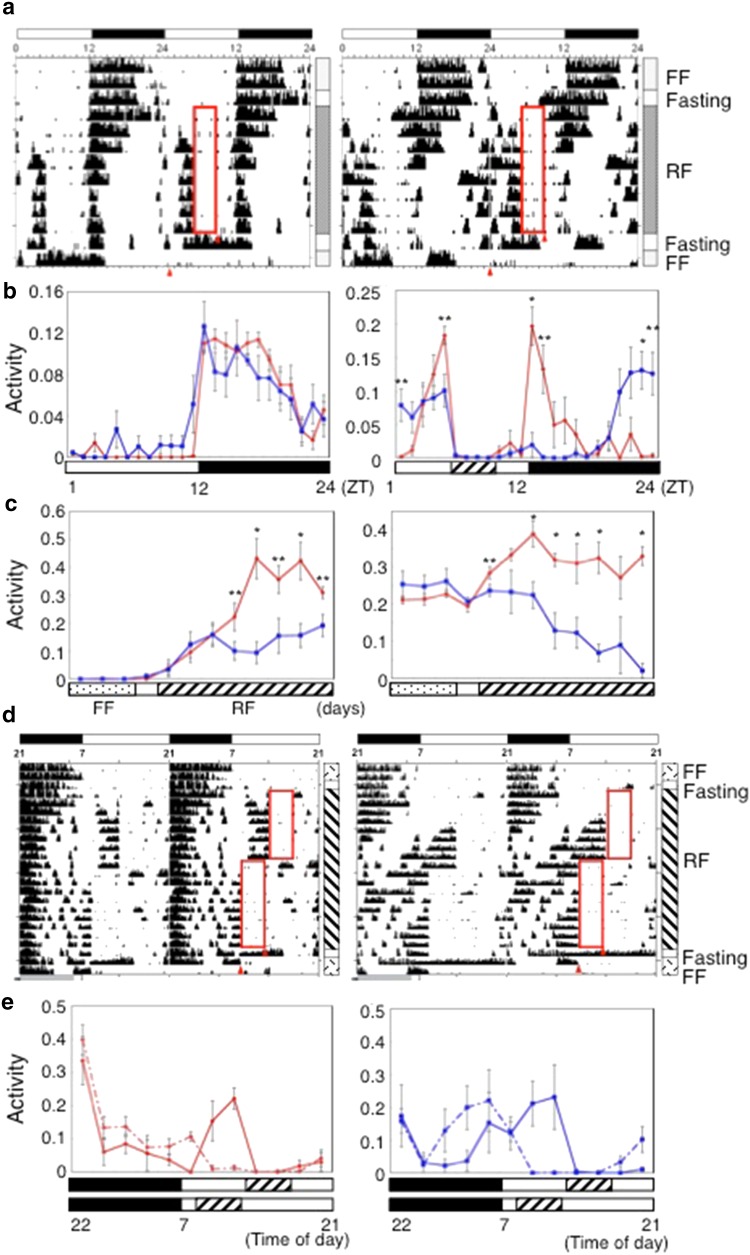
Locomotor activity of the mice under RF in LD conditions. **a** Representative double-plotted actograms of the mice under RF in LD 12:12 condition (LD-RF) for a wild-type mouse (*left panel*) and for a Tg mouse (*right panel*). The *bar* on the *top* of the actogram represents the light (*white*) and dark (*black*) phase of LD 12:12. The *horizontal scale* on each actogram shows ZT. The *bar* on the *right-hand side* of the frame denotes the feeding condition with labels. *Red triangles* in the actograms show the beginning time and the end time of food deprivation during the second fasting following LD-RF. In the actogram, the food available time for 4 h from ZT 5–9 during RF is shown in a *red rectangle*. **b** Activity profiles (*left*, profiles on the day 2 of LD-FF; *right*, profiles on the eighth day of LD-RF). For both Tg mice and WTs (Tg mice, *blue*; WTs, *red*), each data point represents accumulated activity for 1 h at respective ZT, normalized to the total activity for 24 h. *Vertical bar* attached to each data point represents SE (Tg mice, *n* = 7; WTs, *n* = 6). Asterisks show that the difference in the activity level between Tg and WTs is significant (**P < 0.01, *P < 0.001, *t* test). The *bar* at the *bottom* of the profile shows the food available time (*slash*) during RF as well as the light (*white*) and dark (*black*) phase of LD 12:12 labeled with ZT. **c** Course of the daily activity of mice during RF in LD 12:12 condition over 12 days (*left panel*, course of daily pre-feeding activity level; *right panel*, course of daily LD-entrained activity level). In **c**, the relative daily activity levels of pre-feeding activity level (time interval of ZT 3–5) and those of LD-entrained component (time interval of ZT 12–14), normalized to the total activity for 24 h for each genotype group (Tg mice, *blue*; WTs, *red*). The *bar* at the *bottom* of the graph indicates daily feeding conditions. *Vertical bar* attached to each data point represents SE (Tg mice, *n* = 7; WTs, *n* = 6). *Asterisks* show that the difference in the activity level between Tg and WTs is significant (**P < 0.05, *P < 0.01, *t* test). **d** Representative double-plotted actograms of the mice under phase-shift RF schedule in LD 14:10 condition for a wild-type mouse (*left panel*) and for a Tg mouse (*right panel*). *Horizontal scale* in the actogram shows the laboratory time. The *bar* on the *right-hand side* of the frame denotes feeding conditions. The *bar* on the *top* of the actogram shows the light (*white*) and dark (*black*) phases of LD 14:10. The *bar* on the *right-hand side* of the actogram shows feeding conditions labeled as in **a**. Food available time for 4 h during the first and advanced LD-RF is shown in red rectangles in the actogram. *Red triangles* in the actograms denote the beginning and the end time of food deprivation during the second fasting following advanced LD-RF. **e** Activity profiles corresponding to **d** in LD 14:10 (*left panel* WTs; *right panel* Tg mice). In each panel, a *solid line* shows the profile of the eighth day of the first LD-RF. The *dotted line* shows the profile of the eighth day of the advanced LD-RF. For both Tg mice (shown in *blue*) and WTs (shown in *red*), each data point represents accumulated activity for 2 h time interval, normalized to the total activity for 24 h. The bars at the bottom of the profile show the food available time (*slash*) during LD-RF as well as the light (*white*) and dark (*black*) phase of LD cycle for each RF condition scaled with laboratory time. *Vertical bars* attached to each data point represent SE (Tg mice, *n* = 4; WTs, *n* = 7)

To examine the effects of overexpression of C414A mCRY1 on entrainment to RF, we monitored the wheel-running activity of mice under the RF schedule following free feeding (FF) in LD 12:12. Figure 1a portrays typical actograms of both WT and Tg mice. The mean activity profiles of both genotypes are shown in Fig. 1b. The sequence of feeding conditions was as follows: food was available ad libitum under LD 12:12 (LD-FF), then food was deprived for about 1 day (for 27 h) (LD-fasting before LD-RF). Subsequently RF was administered for 8 days during which food was available for 4 h per day (LD-RF); second fasting followed.

During LD-FF, WTs showed normally entrained daily pattern of activity, initiating their nightly bouts of activity at the beginning of the dark period (Fig. 1a, b (left)). Although Tg mice showed, as a whole, a similar pattern of activity to that of WTs during LD-FF, the amount of activity in the light phase in Tg mice was slightly larger than that of WTs (Fig. 1b, left), which reconfirms our earlier result [11]. During LD-RF, food is available only for 4 h from ZT5 to ZT9 (ZT 0, time of light-on; ZT 12, time of light-off). During LD-RF, the activity bouts characteristic of FAA were observed in WTs (Fig. 1a, left). As this actogram clarifies, the emergence of FAA caused a decrease in nocturnal activity in the middle of the night and late night. A similar trend in the temporal change of nocturnal activity during LD-RF was observed previously [12]. Nevertheless the bouts of activity at the beginning of the dark period were maintained throughout LD-RF days in WTs. We designate these components at the beginning of nocturnal activity as LD-entrained activity. In WTs, the activity profile on the eighth day of RF showed a sharp peak corresponding to FAA in addition to the LD-entrained activity (Fig. 1b, right). In the FAA, a peak was located ZT4-5: during 1 h before the food available time. The onset time of FAA was about 4 h before food available time. The FAA has the duration of about 4 h. In WTs, the bouts of activity subsequent to the second LD-fasting occurred simultaneously as the day before during LD-RF. These results demonstrate that WTs showed typical FAA that is driven by typical FEO.

In Tg mice, LD-entrained activity immediately after lights off attenuated with the lapse of LD-RF days. Eventually almost the only activity before the food available time was observed (Fig. 1a, right). Tg mice showed no typical FAA as observed in WTs. Instead, Tg mice displayed a markedly broader band of activity before the food available time during LD-RF: their duration was about 10 h (Fig. 1a [right], b [right]). The activity onset was located around ZT20, which is long before the beginning of the food available time (ZT5). These findings imply that, in Tg mice, RF-entrained activity was generated by self-sustained timing mechanisms that are distinct from FEO working in WTs. To distinguish this mode of activity observed in Tg mice from FAA of WTs, we tentatively designate this component as RF-entrained activity.

Figure 1c presents the course of daily levels of pre-feeding activities (left panel) and LD-entrained activity (right panel) over 8 days. Results show that WTs displayed FAA of a level higher than the pre-feeding activity of Tg mice (Fig. 1c [left]). In WTs the level of the nocturnal LD-entrained activity persisted during RF days (Fig. 1c, right). In Tg mice, with the lapse of RF days, in conjunction with the emergence of RF-entrained activity, the nocturnal LD-entrained activity severely attenuated [Fig. 1c, (right), Supporting Information Fig. S1].

### Phase advance experiments of RF cycles

To obtain another feature of circadian oscillators in Tg mice, we further conducted jet-lag type experiments of RF under LD 14:10. Typical actograms in the LD and feeding conditions of mice are presented in Fig. 1d (left panel, WT; right panel, Tg). The sequence of feeding conditions in Fig. 1d is the following: first food was available ad libitum under LD 14:10 (07:00, time of light-on; 21:00, time of light-off; laboratory time) for 2 days (LD-FF). Then mice were deprived of food for about 1 day (for 27 h: first LD-fasting). Subsequently, the first LD-RF was administered in which the food available period was 4 h (13:00–17:00) per day in the light period for 14 h for 8 days (first LD-RF). Then the phase of RF period was advanced by 4.5 h (08:30–12:30), which continued for 10 days (advanced LD-RF). Then the second LD-fasting for about 2 days (for 44 h) was followed. Activity profiles of mice at the respective eighth days for the first and for the phase advanced LD-RF are shown (Fig. 1e). WTs showed distinct FAA during RF under LD 14:10 (Fig. 1d, left). FAA of WTs re-entrained to the 4.5-h-shifted RF with transient interval. During the second fasting following advanced RF, the consecutive bout of activities corresponding FAA persisted. Distinct LD-entrained activity was sustained during LD-RF (Fig. 1d, left), the same feature as that observed in the LD 12:12 condition (Fig. 1c, right). The LD-entrained activity was disturbed only slightly by the advance of RF cycle (Fig. 1d (left), e (left)]. These results suggest that, in WTs, FEO, which is independent of the SCN, was entrained to the advance of RF, and that the SCN was unaffected by the phase-change of RF. Tg mice showed a broad profile of activity before mealtime during LD-RF also in LD 14:10 [Fig. 1d, e (right)]: this RF-entrained activity, a feature of Tg mice, reflects the situation of the SCN clock as described above. In Tg mice, the phase of RF-entrained activity was shifted in response to the shift of the food available time (Fig. 1d, e [right]). In Tg mice, similar to the case of LD 12:12 (Fig. 1a [right], c [right]), with the lapse of LD-RF days LD-entrained activity severely attenuated (Fig. 1d, right). In the advanced LD-RF the attenuation of LD-entrained activity was also discernible. In the second fasting following advanced RF, the components of RF-entrained activity persisted in the similar phase, which was observed after the advanced RF (Fig. 1d, right).

### Food-entrained circadian rhythm in DD

**Fig. 2:**
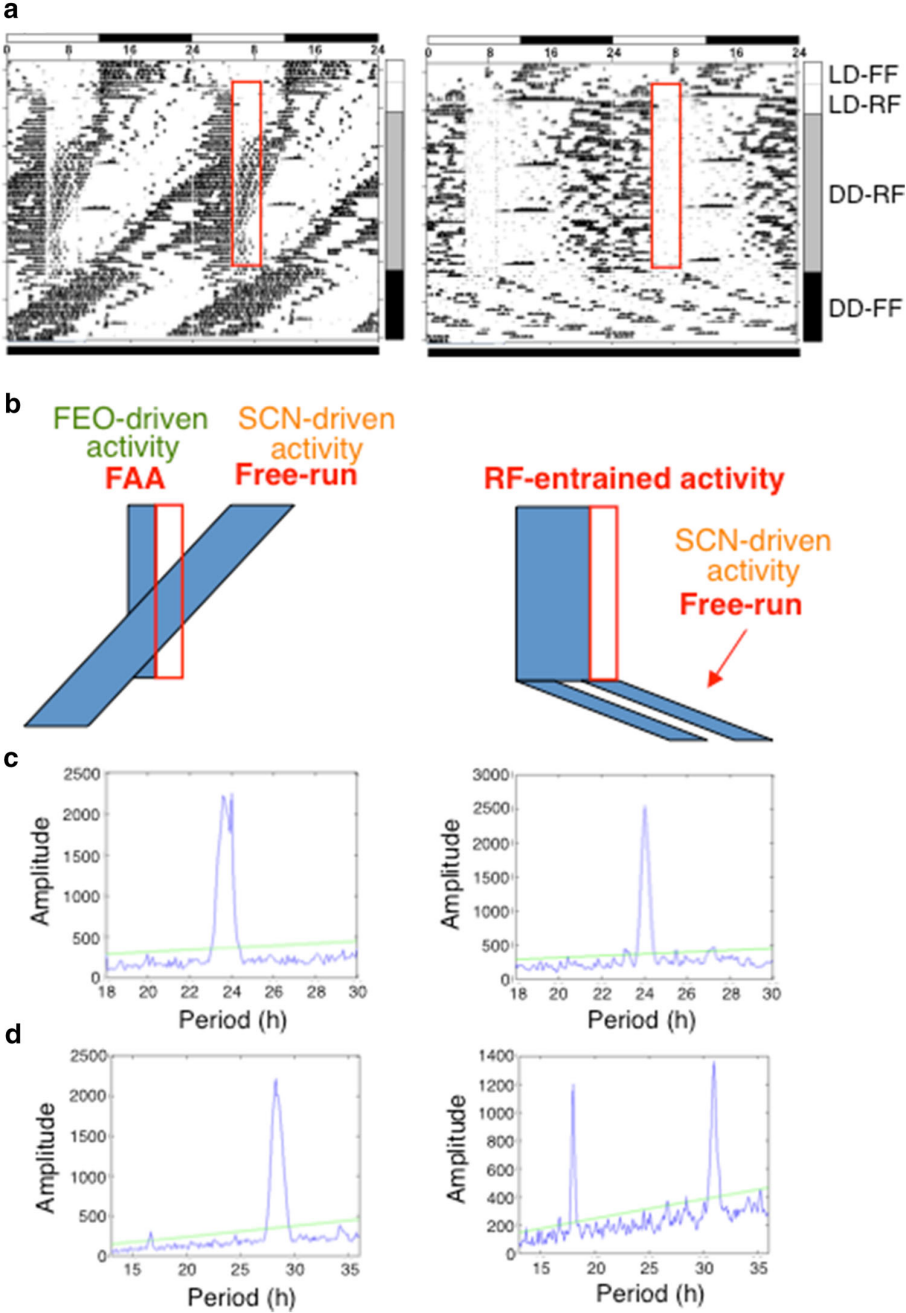
Locomotor activity of the mice under RF in DD condition. Representative wheel-running activity of the mice under RF as well as free feeding in DD condition for a WT mouse (*left*) and for a Tg mouse (*right*). The *horizontal scale* in the actograms shows ZT. The *bar* on the *right-hand side* of each frame denotes the feeding condition with labels. *Bars* on the *top* and the *bottom* of the actograms show the LD 12:12 and DD conditions. In each actogram, the food available time for 4 h is shown in a *red rectangle*. **b** A schematic representation of activity in DD of each actogram (*left panel* WT mouse; *right panel* Tg mouse). The food available time for 4 h during DD-RF is shown in a *red rectangle* also in the schematic diagrams. Activity bands are colored in *blue*. **c** Periodograms under DD-RF. **d** Periodograms under DD-FF. In **c** and **d**, the *left panels* show data for the WT mouse and *right panels* show data for the Tg mouse

We examined locomotor activity under RF in DD condition in detail (Fig. 2a). The sequence of feeding conditions in Fig. 2a was as follows: first food was available ad libitum in LD 12:12 (LD-FF), then food was deprived for about 1 day (27 h) (LD-fasting: label not indicated in Fig. 2a). Subsequently RF was administered for 8 days in which food was available for 4 h per day in LD (LD-RF). Then the light environment was changed to DD, whereas the RF schedule was continued for 41 days (DD-RF), which was followed by an ad libitum feeding condition in DD (DD-FF). Representative actograms of the mice are presented in Fig. 2a [left, wild-type mouse (WT); right, Tg mouse]. All the mice (WTs, *n* = 6; Tg mice, *n* = 5) used in the experiments of this RF regimen showed fundamentally the same activity patterns as that presented in Fig. 2a. As for Tg mice, the data of another mouse are also shown in Supporting Information Fig. S4. The schematized activity patterns in the mice corresponding to the regions of DD-RF and DD-FF in each actogram are presented in Fig. 2b. The periodograms in DD-RF and DD-FF are shown, respectively, in Fig. 2c, d. In the WT, under LD-RF, both FAA and LD-entrained activity were observed (Fig. 2a, left), which is fundamentally the same activity pattern as that presented in Fig. 1a. When the WT mouse was transferred to DD-RF from LD-RF, free-running started from the phase of LD-entrained activity in LD-RF (Fig. 2a, left). This result suggests that the LD-entrained activity observed in LD-RF is driven by the SCN. Throughout DD-RF, FAA persisted; free-running continued independently of FAA (Fig. 2a, b [left] panels). Therefore, during DD-RF, locomotor circadian rhythms of two kinds directed by independent oscillators were observed in WTs. The periodogram displayed two peaks in DD-RF (Fig. 2c, left): one corresponds to FAA (period 24 h). The other corresponds to free-running (period 23.5 h). Shortly after transfer to DD-FF from DD-RF, FAA degenerated, but free-running continued (Fig. 2a, left). In Tg mice, under LD-RF, with the lapse of days, the nocturnal LD-entrained activity almost attenuated, and broad RF-entrained activity was observed (Fig. 2a, right). Consequently, these results confirm the result presented in Fig. 1. In DD-RF, Tg mice showed only one rhythm corresponding to *RF*-*entrained activity* (Fig. 2a, b [right]). They showed no free-running rhythm during DD-RF. In agreement with the results, the periodogram in DD-RF displayed only one peak, which has the same period as that of RF (24 h) (Fig. 2c, right). After returning to DD-FF, the free-running in DD, which is known to be governed by the SCN clock, started from the RF-entrained phase in DD-RF (Fig. 2a, b [right]). The periodogram analysis in DD-FF revealed that this free-running rhythm shares a characteristic feature with our Tg mice: a long period with a split component [11]. These results suggest that the SCN clock was entrained completely to RF in Tg mice under DD-RF.

### Expression of clock genes and clock controlled genes

**Fig. 3:**
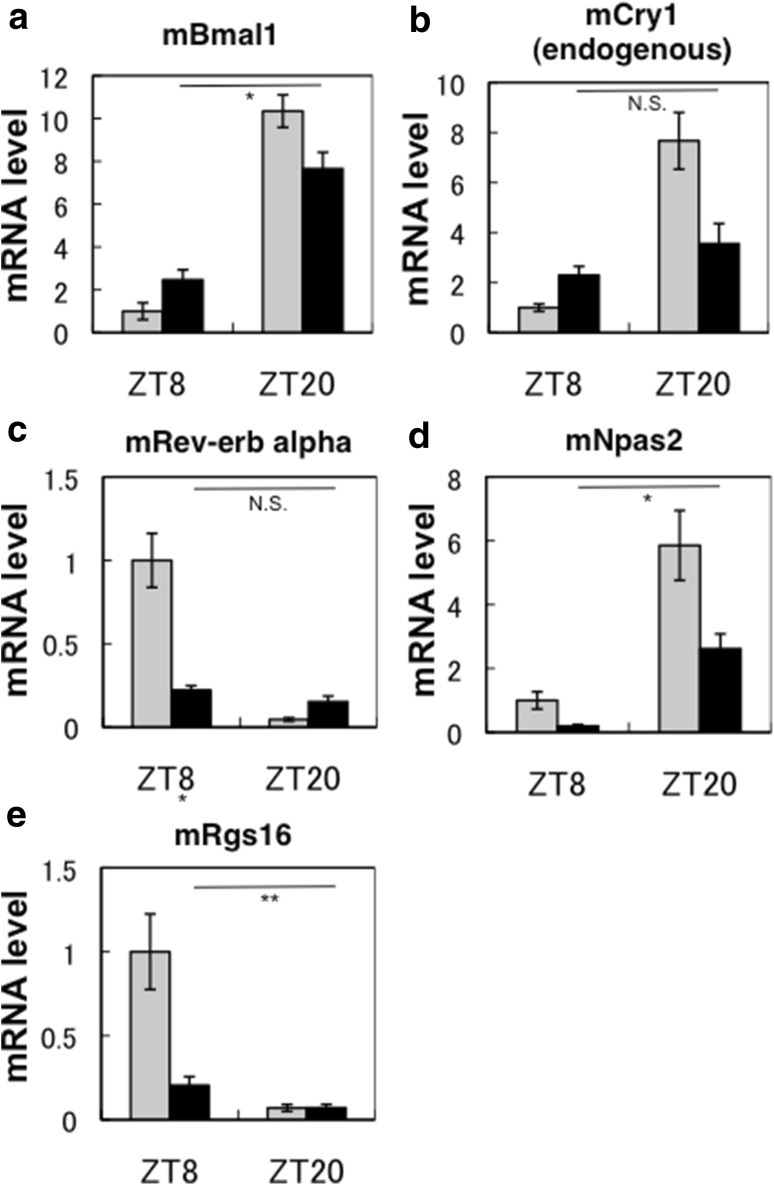
mRNA expression levels in the liver. mRNA expression of clock genes in the liver at ZT 8 and ZT 20 in mice (**a** mBmal1; **b** endogenous mCRY1; **c** mRev-erb alpha; **d** mNpas2; **e** mRgs16). In each representation (*gray bars* WTs; *solid bars* Tg mice), the value for the WTs at ZT 8 was set to 1. The *vertical bar* attached to each data point represents SE (*n* = 7–12). *Asterisks* show that the respective differences in the expression level between ZT8 and ZT 20 in Tg mice are significant (**P < 0.05, *P < 0.00001, *t* test). N.S. shows that no significant difference exists between ZT8 and ZT 20 (*t* test). As for WTs, all respective differences in the expression levels between ZT8 and ZT 20 were statistically significant (*P* < 0.001, *t* test)

To elucidate the molecular mechanism of the unusual circadian behaviors in Tg mice presented above, we conducted real-time PCR analyses using cDNA derived from the liver of mice under ad lib feeding at ZT8 and ZT20 in LD 12:12 (Fig. 3). In the same sample we previously demonstrated that the amplitudes of circadian oscillation of mPer2 and mDbp are considerably reduced in Tg mice [11]. As expected, the amplitudes of circadian oscillation of core clock including endogenous mCry1 genes in the liver of Tg mice were quite damped (Fig. 3a–d). In addition, mRgs16 is a clock-controlled gene including E-box and D-box in its upstream region [16]. The amplitude of circadian expression of mRgs16 is markedly suppressed in Tg mice (Fig. 3e).

## Discussion

These studies suggest that the Tg mice overexpressing mutant CRY1 can be entrained to RF-cycle in LD and DD conditions. Results show that Tg mice exhibited distinct food-entrained rhythms from those of WTs. The WTs showed typical FAA, in which the amount of activity bouts sharply rises immediately before meal time, whereas Tg mice show a broader range of pre-feeding activity (RF-entrained activity) without any evident peak in LD or DD (Figs. 1, 2). In Tg mice, LD-entrained activity right after lights off attenuated with the elapse of LD-RF days, and eventually almost the only activity before the food available time was observed (Figs. 1, S1). It is noteworthy that the overall RF-entrained activity in LD-RF continues from the bouts of activity in the dark period in LD-FF (Figs. 1a, S1). These results suggest a possibility that RF-entrained activity in Tg mice is driven by the SCN, and that the SCN clock entrained to RF in Tg mice. Also these results imply that Tg mice were almost entirely entrained to RF even in LD, and that in Tg mice feeding cycle became dominant over LD cycle as an entrainment agent to the SCN clock. In Tg mice, upon transfer to the subsequent LD-fasting following LD-RF, the onset time of activity was almost identical as the day before under RF condition (Figs. 1a [right], S1). This result implies that the oscillation of SCN clock free-runs in the absence of RF zeitgeber in Tg mice. In addition, the results of phase advance experiments of RF cycles suggest that the SCN clock in Tg mice is re-entrainable to the phase-advance of RF (Fig. 1d, e).

Figure 3 and previous study [11] show that the amplitudes of circadian oscillation of various clock genes in the liver of Tg mice were quite damped. These results also show agreement with recent reports from other groups showing that C414A mCRY1 works as a stronger repressor than wild-type mCRY1 to the CLOCK-BMAL1-induced transcription in *Cry*-deficient mouse embryo fibroblast (MEF) cells [10, 17]. In agreement with the results, using the newly created C414A mCRY1-overexpressing HEK293 cells, we demonstrated that C414A mCRY1 has stronger repressive activities to E-box controlled clock genes than intact mCRY1 does in human cells (Supporting Information Fig. S3). Importantly, C414A mCRY1 introduced *Cry*-deficient MEF cells showed a longer period of circadian rhythm than that of wild-type mCRY1 introduced cells [17], which is consistent with our in vivo measurements of free-running periods in Tg mice [11].

Based on these accumulated results, we reason that the amplitude of circadian oscillation is dampened in each single neuronal level in the SCN of Tg mice, and that the weakened amplitude of cellular molecular clock in each neuron in the SCN can be accountable for the aberrant entrainment behavior of the SCN. To support this idea, mice having weakened cellular clockwork are reported to exhibit altered FAA in DD [18]. *Per2*^Brdm1^/*Cry2*^−*/*−^ double mutant mice are particularly similar to our phenotypes: they entrain to RF in DD [18], suggesting that the SCN of *Per2*^Brdm1^/*Cry2*^−*/*−^ mutant mice entrain to RF in DD. The mice also show long-lasting pre-feeding activities similar to RF-entrained activity in our Tg mice [18]. Moreover *Per2*^Brdm1^/*Cry2*^−*/*−^ mutant mice can re-entrain to the phase-shift of RF cycle [18] as our Tg mice do (Fig. 1). Further, Erzberger et al. showed that *Per2*^Brdm1^/*Cry*2^−/−^ is able to entrain to the extreme range of T cycles of LD [19]. Based on the theory of synchronization of limit cycle oscillations to external zeitgebers, in conjunction with the experimentally obtained results, they inferred that weakened clock enables mice to entrain to aberrant range of zeitgeber cycles [19]. In accordance with the mathematical model, Takasu et al. demonstrated that *Bmal*1 knock-out mice, in which molecular clockwork is expected to be weakened in each cellular level, show an extreme range of T-cycles of RF [20]. Taken together, the weakened amplitude of cellular molecular clock in each neuron in the SCN can be regarded as a primary cause for the aberrant entrainment behavior of the SCN, such as the entrainment of the SCN to RF cycles. Our results suggest a possibility that, in Tg mice, the RF cycle works even more strongly than the LD cycle does as an entrainment agent, which is not the case for WTs (Figs. 1, S1).

The coupling defect might also cause the loss of an intrinsic robustness of SCN to external input from feeding. As a consequence, the SCN clock in Tg mice entrains to RF cycle. Recent reports have demonstrated that evidence exists to show that the cellular couplings in the SCN of neonatal and adult mice differ. Reportedly, the SCN of mice in fetal stages entrains to maternal feeding timing, indicating that the neonatal SCN is entrainable to non-photic time cues [21]. Based on detailed analyses conducted using *Cry*1^−/−^/*Cry*2^−/−^ mice of both neonatal and adult stages, Ono et al. reported that the reorganization in the coupling of the SCN occurs during the developmental process, and that CRY proteins are involved in the changes of the coupling mode of SCN neurons from the neonatal type to the adult type [22]. Consequently, the possibility exists for our Tg mice that the overexpression of C414A mCRY1 influences the development of cell networks of SCN and hinders the development of the mode of cellular couplings from the neonatal type to the adult type. Consequently, still in adult Tg mice, the SCN has the same characteristics of the neonatal characters, thereby being entrainable to non-photic feeding of a zeitgeber cycle. A promising cause of possible differences in the organization of the SCN between Tg and WTs is the possible reduction of mRgs16 in each SCN neuron in Tg mice, which can alter the coupling [16]. As for the *Per2*^Brdm1^/*Cry2*^−*/*−^ mutant mice described above, reportedly when RF is conducted in LD, the mice showed persistent LD-entrained activity without displaying FAA during LD [18]. These results suggest that, in LD-RF, the SCN of *Per2*^Brdm1^/*Cry2*^−*/*−^ mutant mice continue entraining to the LD cycle and do not respond to RF. The different responses to RF from our Tg mice may be due to difference in coupling modes. Results showed that WTs free-run with long free-running periods in constant light with *ad lib* feeding (LL-FF; Supporting Information Fig. S2), which agrees with results described in earlier reports [23]. Whereas Tg mice free-run with short periods in LL-FF (Fig. S2). Tg mice also showed long-lasting prefeeding activity bouts under RF in LL (Fig. S2). Importantly, the phases from which free-running starts in DD-FF are apparently coincide with those of LL-RF (Fig. S2), which is the same feature as in the case from DD-RF to DD-FF in Tg mice (Fig. 2a, b [right]; see also Fig. S4), suggesting that the SCN clock entrained to RF in Tg mice also in LL. Recent study suggested that LL has modifying effects on the SCN coupling [23, 24]. The opposite effects of LL on the free-running period of WTs and Tg mice (Fig. S2) may reflect the difference in the mode of cellular couplings in the SCN between Tg mice and WTs.

In summary, although yet another interpretation of the unique food-entrained behavior of Tg mice by hourglass mechanism [25] associated with putative hypersensitivity to hunger can not entirely be excluded, many related findings of ours and of others point to the fact that the overexpression of Cys414-Ala mutant mCRY1 weakens both the interneuronal coupling and the intraneuronal oscillation of the SCN, which are typically manifested in the easy entrainment to the feeding cycle, as well as in the fast re-entrainment to the shift of LD cycles.

## Electronic supplementary material

Below is the link to the electronic supplementary material.

Supplementary material 1 (DOC 41 kb)

Supplementary material 2 (TIFF 2931 kb)

Supplementary material 3 (TIFF 2931 kb)

Supplementary material 4 (TIFF 2931 kb)

Supplementary material 5 (TIFF 2931 kb)

## References

[1] Alvarez JD, Sehgal A (2004). Genetic basis for circadian rhythms in mammals. Molecular biology of circadian rhythms.

[2] van der Horst GT, Muijtjens M, Kobayashi K, Takano R, Kanno S, Takao M, de Wit J, Verkerk A, Eker AP, van Leenen D, Buijs R, Bootsma D, Hoeijmakers JH, Yasui A (1999). Mammalian Cry1 and Cry2 are essential for maintenance of circadian rhythms. Nature.

[3] Honma S, Ono D, Suzuki Y, Inagaki N, Yoshikawa T, Nakamura W, Honma K (2012). Suprachiasmatic nucleus: cellular clocks and networks. Prog Brain Res.

[4] Welsh DK, Takahashi JS, Kay SA (2010). Suprachiasmatic nucleus: cell autonomy and network properties. Annu Rev Physiol.

[5] Buhr ED, Yoo SH, Takahashi JS (2010). Temperature as a universal resetting cue for mammalian circadian oscillators. Science.

[6] Abraham U, Granada AE, Westermark PO, Heine M, Kramer A, Herzel H (2010). Coupling governs entrainment range of circadian clocks. Mol Syst Biol..

[7] Yamaguchi Y, Suzuki T, Mizoro Y, Kori H, Okada K, Chen Y, Fustin JM, Yamazaki F, Mizuguchi N, Zhang J, Dong X, Tsujimoto G, Okuno Y, Doi M, Okamura H (2013). Mice genetically deficient in vasopressin V1a and V1b receptors are resistant to jet lag. Science.

[8] Mieda M, Ono D, Hasegawa E, Okamoto H, Honma K, Honma S, Sakurai T (2015). Cellular clocks in AVP neurons of the SCN are critical for interneuronal coupling regulating circadian behavior rhythm. Neuron.

[9] Kon N, Yoshikawa T, Honma S, Yamagata Y, Yoshitane H, Shimizu K, Sugiyama Y, Hara C, Kameshita I, Honma K, Fukada Y (2014). CaMKII is essential for the cellular clock and coupling between morning and evening behavioral rhythms. Genes Dev.

[10] Schmalen I, Reischl S, Wallach T, Klemz R, Grudziecki A, Prabu JR, Benda C, Kramer A, Wolf E (2014). Interaction of circadian clock proteins CRY1 and PER2 is modulated by zinc binding and disulfide bond formation. Cell.

[11] Okano S, Akashi M, Hayasaka K, Nakajima O (2009). Unusual circadian locomotor activity and pathophysiology in mutant CRY1 transgenic mice. Neurosci Lett.

[12] Nakamura W (2010). Behavioral analysis of circadian rhythms: entraining the circadian clock and determining the food-entrainable oscillator mechanism. Sleep Biol Rhythms..

[13] Abe H, Honma S, Honma K (2007). Daily restricted feeding resets the circadian clock in the suprachiasmatic nucleus of CS mice. Am J Physiol Regul Integr Comp Physiol.

[14] Okano S, Hayasaka K, Igarashi M, Iwai H, Togashi Y, Nakajima O (2010). Non-obese early onset diabetes mellitus in mutant cryptochrome1 transgenic mice. Eur J Clin Invest.

[15] Okano S, Hayasaka K, Igarashi M, Togashi Y, Nakajima O (2013). Characterization of age-associated alterations of islet function and structure in diabetic mutant cryptochrome 1 transgenic mice. J Diabetes Investig..

[16] Doi M, Ishida A, Miyake A, Sato M, Komatsu R, Yamazaki F, Kimura I, Tsuchiya S, Kori H, Seo K, Yamaguchi Y, Matsuo M, Fustin JM, Tanaka R, Santo Y, Yamada H, Takahashi Y, Araki M, Nakao K, Aizawa S, Kobayashi M, Obrietan K, Tsujimoto G, Okamura H (2011). Circadian regulation of intracellular G-protein signalling mediates intercellular synchrony and rhythmicity in the suprachiasmatic nucleus. Nat Commun..

[17] Nangle SN, Rosensweig C, Koike N, Tei H, Takahashi JS, Green CB, Zheng N (2014). Molecular assembly of the period-cryptochrome circadian transcriptional repressor complex. Elife..

[18] Mendoza J, Albrecht U, Challet E (2010). Behavioural food anticipation in clock genes deficient mice: confirming old phenotypes, describing new phenotypes. Genes Brain Behav..

[19] Erzberger A, Hampp G, Granada AE, Albrecht U, Herzel H (2013). Genetic redundancy strengthens the circadian clock leading to a narrow entrainment range. J R Soc Interface.

[20] Takasu NN, Kurosawa G, Tokuda IT, Mochizuki A, Todo T, Nakamura W (2012). Circadian regulation of food-anticipatory activity in molecular clock-deficient mice. PLoS ONE.

[21] Ohta H, Xu S, Moriya T, Iigo M, Watanabe T, Nakahata N, Chisaka H, Hanita T, Matsuda T, Ohura T, Kimura Y, Yaegashi N, Tsuchiya S, Tei H, Okamura K (2008). Maternal feeding controls fetal biological clock. PLoS ONE.

[22] Ono D, Honma S, Honma K (2013). Cryptochromes are critical for the development of coherent circadian rhythms in the mouse suprachiasmatic nucleus. Nat Commun..

[23] Ohta H, Yamazaki S, McMahon DG (2005). Constant light desynchronizes mammalian clock neurons. Nat Neurosci.

[24] Ono D, Honma S, Honma K (2013). Postnatal constant light compensates Cryptochrome1 and 2 double deficiency for disruption of circadian behavioral rhythms in mice under constant dark. PLoS One.

[25] Hiroshige T, Honma K, Honma S (1991). SCN-independent circadian oscillators in the rat. Brain Res Bull.

