# Supplementary material for: Unique food-entrained circadian rhythm in cysteine414-alanine mutant mCRY1 transgenic mice
Source: Sleep Biol Rhythms. 2016 Jan 29;14:261–9. doi: 10.1007/s41105-016-0050-1 (PMC4932127; doi:10.1007/s41105-016-0050-1)
Supplement: Supplementary file 1 — Supplementary material 1 (DOC 41 kb) [file 41105_2016_50_MOESM1_ESM.doc]

**Supporting information**

**Supplementary materials and methods**

**Primer sets used for Real-Time PCR**

Primer sets for mice are: mHprt-5': GGACCTCTCGAAGTGTTGGATACAGG, mHprt-3': CTGGCAACATCAACAGGACTCCTCGT, mBmal1-5': GAAAGGCAGTGCCACTGACTACCAA, mBmal1-3': GAACAGCCATCCTTAGCACGGTGAG, mNpas2-5': CGGGACCAGTTCAATGTTCT, mNpas2-3': CCATCTAATGCCTCCAACAT

mRev-erb alpha-5': CTTCCGTGACCTTTCTCAGC, mRev-erb alpha-3': CAGCTCCTCCTCGGTAAGTG, mRgs16-5': TCAGAGCTGAGCTCCGATACTGGGG, mRgs16-5': GCCAGTTTGGTGGCTGATCGGATCT, mCry1-5': AGGAGGTTTTCTTGGAAGCGCAGGT, mCry1-3': CGAGGATATAGACGCAGCGGATGGT

Primer sets for human are: hHprt-5': ACCCCACGAAGTGTTGGATATAAGCCAGAC, hHprt-3': TGGCGATGTCAATAGGACTCCAGATGTTTCC, hCry1-5': CCCAGGTTGTAGCAGCAGTGGAAG, hCry-3': CACTGAGACCAGTGCCCATGGAG, hPer2-5': GCAGTGACTGTGACGACAGTGGGA, hPer2-3': TACTGGCCTTGCCCTTGGCCTTCTTG

**Quantification of mRNA in HEK293 by Real-Time PCR**

　mCRY1 cDNA with or without C414A mutation was attached at its amino terminus with FLAG and was transfected into HEK293 using Flp-In system (Invitrogen Corp.) to obtain a cell line inducibly expressing FLAG-mCRY1 or FLAG-C414A mCRY1. For the controls, the HEK293 cell line was also created, in which empty vector was introduced. The expression levels of hCry1 and hPer2 mRNA were examined in HEK293 cells 48 h after the administration of doxycycline. The expression of FLAG-mCRY1s in each HEK293 was equivalent level in the induction condition (data not shown). cDNA synthesis and real-time PCR analysis were conducted as described in *Materials and Methods*. hHPRT was used as the endogenous control for quantification.

**Food-entrained circadian rhythm in LL**

We performed RF experiments of WT and Tg mice in the LL condition. The sequence of the schedule was as follows: mice were kept in LD 12:12 with *ad libitum* feeding for more than 1 month. Then the mice were transferred to LL with *ad libitum* feeding (LL-FF) and kept for more than 1 month. The mice were deprived of food for 27 h (LL-fasting). Subsequently, RF under LL was administered in which food was available 4 h (12:00–16:00; laboratory time) per day for 8 days (LL-RF), then transferred to DD with *ad libitum* feeding (DD-FF). The results are shown in Fig. S1.

In LL-FF WTs free-run with long periods (A, B, left panels; D, right panel). To the contrary Tg mice free-run with short periods in LL-FF (A, B, D, right panels); and the free-run activity in LL-FF did not split (A, B, right panels). Some animal individuals became arrhythmic in LL-FF (actograms not shown: wild type, 1 individual out of 9; Tg, 2 individuals out of 9). In WTs, typical FAA with sharp peak was observed also in LL-RF (A, left panel). In Tg mice, instead of typical FAA, distinct *RF-entrained activity* was observed in LL-RF (A, right panel). Activity bands before the food available time in Tg mice were considerably broader than that of WTs during LL-RF (E, left panel). Upon transfer to DD-FF from LL-RF, both WTs and Tg mice started free-running again (A). The periodogram of Tg mice under DD-FF displays two peaks: a long free-running period and an additional high peak at one-half of the free-running period corresponding to the split component (C, right panel).

**Supplementary discussion**

Results show that WTs free-run with long free-running periods in constant light with *ad lib* feeding (LL-FF; Fig S1), and some individuals showed arrhythmicity. These features are in consistent with results described in earlier reports: by the exposure of LL to rodents, their free-running periods were lengthened, and some individuals became arrhythmic [23, 26]. By contrast to WTs, Tg mice free-run with short periods in LL-FF (Fig. S1). The arrhythmic phenotype in mice under LL is suggested to be attributable to that LL disturbs the coupling among SCN neurons without affecting circadian rhythms of each individual neuron [23]. It is reported that in some clock-gene mutated mice, the restoration of circadian locomotor rhythms by keeping in LL. For instance, *Clock* mutant [27] and *Per2*^Brdm1^/*Cry1^-/-^* mice [28], both of which display arrhythmic phenotype under DD, become rhythmic under LL. Interestingly, the period of the restored circadian rhythms in *Per2*^Brdm1^/*Cry1^-/-^* mice in LL is short [28], as was observed in our Tg mice in LL (Fig. S1)*.* One of attractive explanations of the effects of LL to free-running periods is as follows: the modifying effects of LL on the circadian rhythms are different depending on the organization of the SCN, possibly the mode of coupling among neurons. The opposite effects of LL on the free-running period may reflect the difference in the mode of cellular couplings in the SCN between Tg mice and WTs.

**Supplementary figure legends**

**Figure S1.** Supporting two actograms for Tg mice under RF in LD 12:12 condition.

The feeding schedules are the same as in Fig. 1A.

**Figure S2.** Representative double-plotted actograms of the mice under RF in LL and DD conditions for a WT mouse (left panel) and for a Tg mouse (right panel). The horizontal scale in the actograms shows the laboratory time. The bar on the right-hand side of the frame denotes the feeding condition with labels as follows: LL-FF, FF under LL; LL-fasting, total deprivation of food under LL; LL-RF, RF under LL; DD-FF, FF under DD. The bars on the top and the bottom of the actograms represent respectively the LL and DD conditions. In each actogram, food available time for 4 h from laboratory time 12:00–16:00 during LL-RF is shown in a red rectangle. Red triangles in the actograms indicate the beginning of DD-FF. **(B)** The periodograms of a wild-type mouse (left panel) and Tg mouse (right panel) in LL-FF. **(C)** The periodograms of a wild-type mouse (left panel) and Tg mouse (right panel) in DD-FF. **(D)** Free-running periods of Tg as well as WTs in the respective DD-FF (left panel: WTs, *n*=9 [23.65±0.05 h]; Tg mice, *n*=9 [28.12±0.61 h], * P = 2.54E-07, *t*-test, comparison between WTs and Tg mice) and LL-FF (right panel: WTs, *n*=8 [25.31±0.21 h]; Tg mice, *n*=7 [21.36±0.42 h], * P = 1.15E-06, *t*-test, comparison between WTs and Tg mice) conditions. Bars represent SE. The left panel in **(E)** presents the activity profiles on the seventh day of LL-RF. For both Tg mice (blue) and WTs (red), each data point represents accumulated activity for 2 h time interval, normalized to the total activity for 24 h. The bar at the bottom of the profile shows the food available time (slash) during LL-RF. The right panel in **(E)** shows the course of daily pre-feeding activity (activity of time interval of laboratory time of 10:00–12:00) of mice during the LL-RF schedule. The bar at the bottom of the graph shows the daily feeding conditions (Tg mice, blue; WTs, red). The vertical bar attached to each data point represents SE (Tg mice, *n*=11; WTs, *n*=13). Asterisks show that the difference in the activity level between Tg and WTs is significant (*** *P* < 0.05, ** *P* < 0.001, * *P* < 0.0001, *t*-test, comparison between WTs and Tg mice).

**Figure S3.** **hCry1 and hPer2 mRNA expression in human HEK293 overexpressing FLAG-mCRY1 or FLAG-C414A mCRY1.**

In each representation, the value for the controls in which empty vector was introduced was set to 1. Each value (grey bars: control, black bars: FLAG-C414A mCRY1, slash bars: FLAG-mCRY1) represents means ± SE (hCry1: *n*=6, hPer2: *n*=5). For intact FLAG-mCRY1 expression, no significant change was observed compared with the controls. In contrast, for FLAG-C414A mCRY1, both hCry1 and hPer2 mRNA levels were reduced significantly (one-way ANOVA, hCry1: F(2,15)=3.68, P=1.07E-07; hPer2: F(2,14)=3.89, P =3.74E- 04, followed by post hoc *t*-test).

**Figure S4.** **Supporting actograms for Tg mice.**

(A) from DD-RF to DD-FF, (B) from LL-RF to DD-FF. The feeding schedules are the same as in Fig. 2 and Fig. S1 respectively.

**Supplementary References**

26. Aschoff J. (1960) Exogenous and endogenous components in circadian rhythms. Cold Spring Harb Symp Quant Biol., 25: 11–28.

27. Spoelstra K, Oklejewicz M, and Daan S. (2002) Restoration of self-sustained circadian rhythmicity by the mutant clock allele in mice in constant illumination. J Biol Rhythms., 17: 520-5.

28. Abraham D, Dallmann R, Steinlechner S, Albrecht U, Eichele G, and Oster H. J (2006) Restoration of circadian rhythmicity in circadian clock-deficient mice in constant light. Biol Rhythms., 21: 169-76.
