# Supplementary figures and images for: Unique food-entrained circadian rhythm in cysteine414-alanine mutant mCRY1 transgenic mice
Source: Sleep Biol Rhythms. 2016 Jan 29;14:261–9. doi: 10.1007/s41105-016-0050-1 (PMC4932127; doi:10.1007/s41105-016-0050-1)

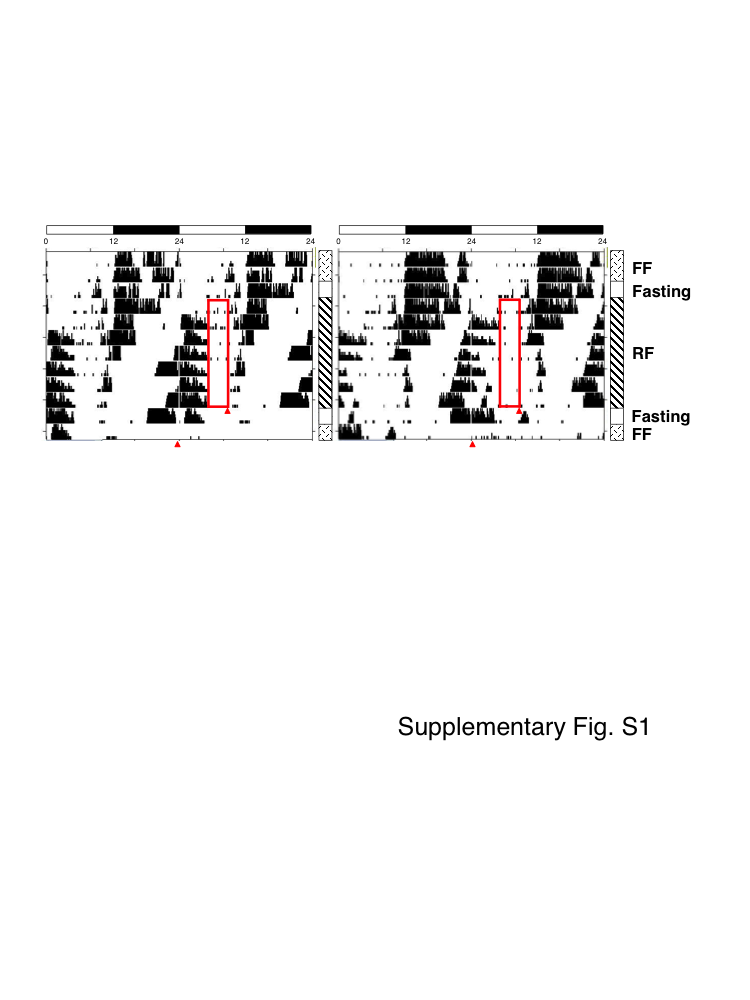

Supplement: Supplementary file 2 — Supplementary material 2 (TIFF 2931 kb) [file 41105_2016_50_MOESM2_ESM.tif]

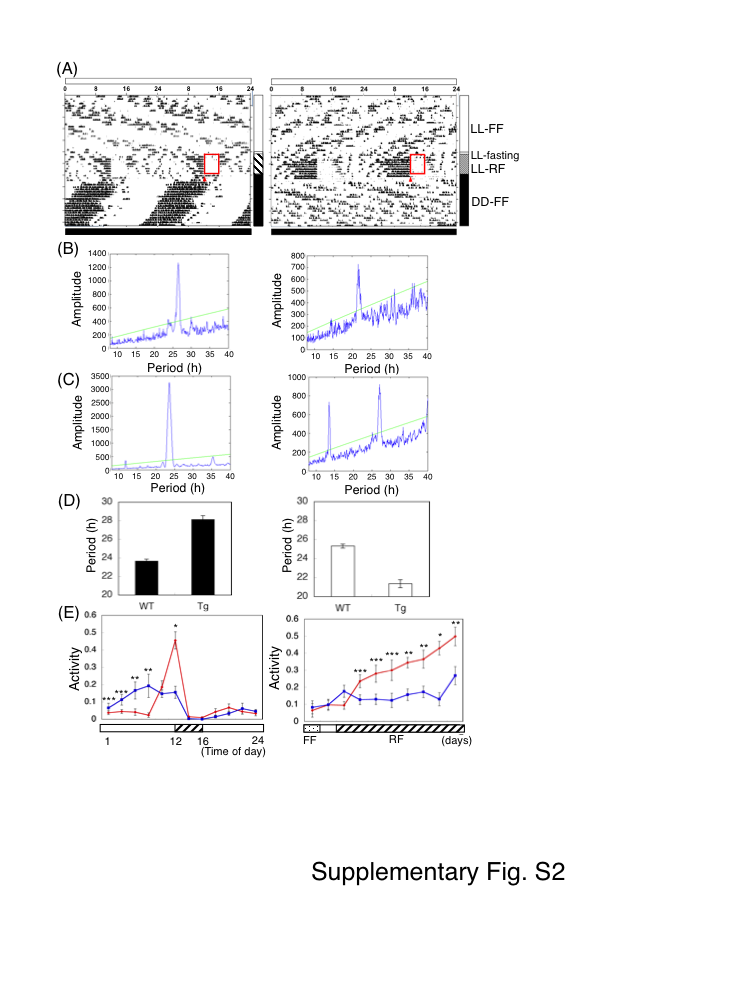

Supplement: Supplementary file 3 — Supplementary material 3 (TIFF 2931 kb) [file 41105_2016_50_MOESM3_ESM.tif]

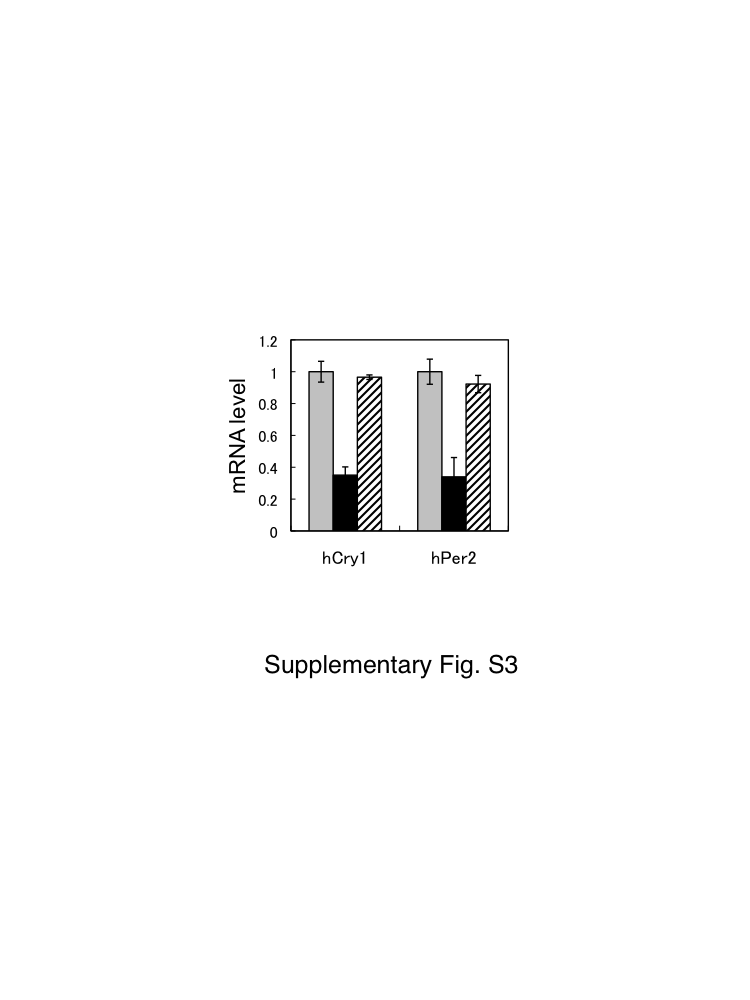

Supplement: Supplementary file 4 — Supplementary material 4 (TIFF 2931 kb) [file 41105_2016_50_MOESM4_ESM.tif]

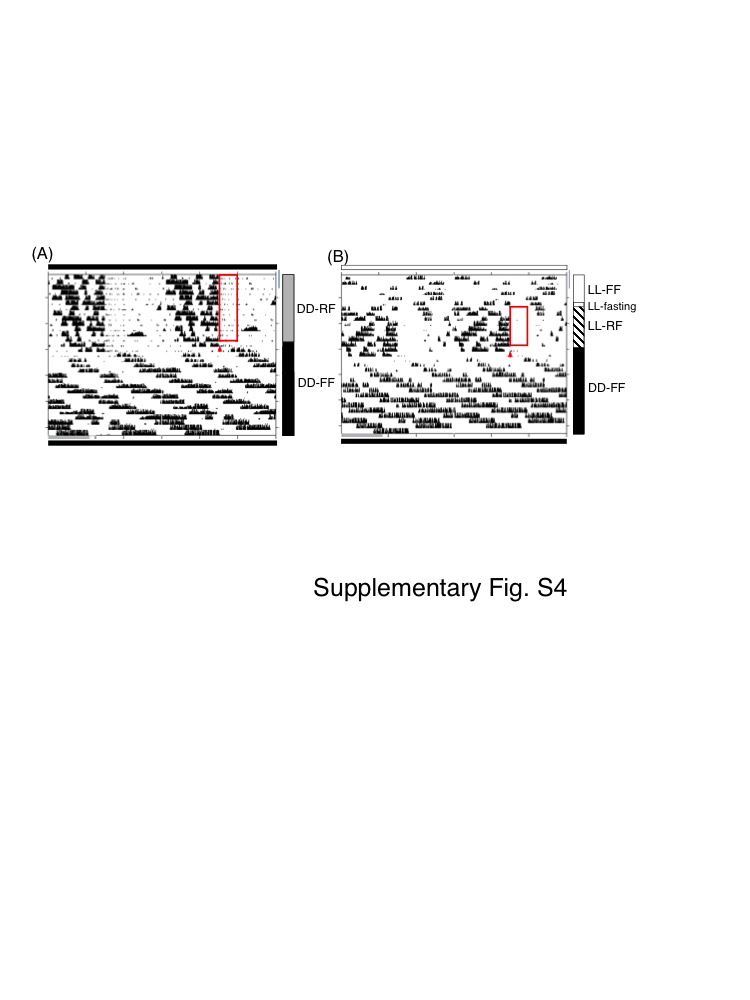

Supplement: Supplementary file 5 — Supplementary material 5 (TIFF 2931 kb) [file 41105_2016_50_MOESM5_ESM.tif]
